# The WATCH-DM risk score estimates clinical outcomes in type 2 diabetic patients with heart failure with preserved ejection fraction

**DOI:** 10.1038/s41598-024-52101-8

**Published:** 2024-01-19

**Authors:** Katsuomi Iwakura, Toshinari Onishi, Atsunori Okamura, Yasushi Koyama, Nobuaki Tanaka, Masato Okada, Kenshi Fujii, Masahiro Seo, Takahisa Yamada, Masamichi Yano, Takaharu Hayashi, Yoshio Yasumura, Yusuke Nakagawa, Shunsuke Tamaki, Akito Nakagawa, Yohei Sotomi, Shungo Hikoso, Daisaku Nakatani, Yasushi Sakata, PURSUIT-HFpEF Investigators, Tetsuya Watanabe, Yoshiharu Higuchi, Masaharu Masuda, Mitsutoshi Asai, Toshiaki Mano, Hisakazu Fuji, Daisaku Masuda, Ryu Shutta, Shizuya Yamashita, Masami Sairyo, Haruhiko Abe, Yasunori Ueda, Yasushi Matsumura, Kunihiko Nagai, Masami Nishino, Jun Tanouchi, Yoh Arita, Nobuyuki Ogasawara, Takamaru Ishizu, Minoru Ichikawa, Yuzuru Takano, Eisai Rin, Yukinori Shinoda, Koichi Tachibana, Shiro Hoshida, Masahiro Izumi, Hiroyoshi Yamamoto, Hiroyasu Kato, Kazuhiro Nakatani, Yuji Yasuga, Mayu Nishio, Keiji Hirooka, Takahiro Yoshimura, Kazunori Kashiwase, Shinji Hasegawa, Akihiro Tani, Yasushi Okumoto, Yasunaka Makino, Yoshiyuki Kijima, Takashi Kitao, Masashi Fujita, Koichiro Harada, Masahiro Kumada, Osamu Nakagawa, Ryo Araki, Takayuki Yamada, Yuki Matsuoka, Taiki Sato, Akihiro Sunaga, Bolrathanak Oeun, Hirota Kida, Tomoharu Dohi, Yasuhiro Akazawa, Kei Nakamoto, Katsuki Okada, Fusako Sera, Hidetaka Kioka, Tomohito Ohtani, Toshihiro Takeda, Hiroya Mizuno

**Affiliations:** 1 Division of Cardiology, Sakurabashi Watanabe Hospital, 2-4-32, Umeda, Kita-ku, Osaka, 5300001 Japan; 2 Department of Cardiovascular Medicine, Sakai City Medical Center, Sakai, Japan; 3 Division of Cardiology, Osaka General Medical Center, Osaka, Japan; 4 Division of Cardiology, Osaka Rosai Hospital, Sakai, Japan; 5 Cardiovascular Division, Osaka Police Hospital, Osaka, Japan; 6 Division of Cardiology, Amagasaki Chuo Hospital, Amagasaki, Japan; 7 Division of Cardiology, Kawanishi City Medical Center, Kawanishi, Japan; 8 Department of Cardiology, Rinku General Medical Center, Izumisano, Japan; 9 Department of Cardiology, Pulmonology, Hypertension & Nephrology, Ehime University Graduate School of Medicine, Toon, Japan; 10 Department of Medical Informatics, Osaka University Graduate School of Medicine, Osaka, Japan; 11 Department of Cardiovascular Medicine, Osaka University Graduate School of Medicine, Osaka, Japan; 12 Cardiovascular Medicine, Nara Medical University, Kashihara, Japan; 13 Kansai Rosai Hospital, Amagasaki, Japan; 14 Kobe Ekisaikai Hospital, Kobe, Japan; 15 National Hospital Organization Osaka National Hospital, Osaka, Japan; 16 Ikeda Municipal Hospital, Ikeda, Japan; 17 Japan Community Health Care Organization Osaka Hospital, Osaka, Japan; 18 Higashiosaka City Medical Center, Higashiosaka, Japan; 19 Kawachi General Hospital, Higashiosaka, Japan; 20 Yao Municipal Hospital, Yao, Japan; 21 Kinki Central Hospital, Itami, Japan; 22 Japan Community Health Care Organization, Osaka Minato Central Hospital, Osaka, Japan; 23 Sumitomo Hospital, Osaka, Japan; 24 Saiseikai Senri Hospital, Suita, Japan; 25 National Hospital Organization Osaka Minami Medical Center, Kawachinagano, Japan; 26 Kano General Hospital, Osaka, Japan; 27 Kinan Hospital, Tanabe, Japan; 28 Hyogo Prefectural Nishinomiya Hospital, Nishinomiya, Japan; 29 Japan Community Health Care Organization, Hoshigaoka Medical Center, Hirakata, Japan; 30 Minoh City Hospital, Minoh, Japan; 31 Osaka International Cancer Institute, Osaka, Japan; 32 Suita Municipal Hospital, Suita, Japan; 33 Toyonaka Municipal Hospital, Toyonaka, Japan; 34 Otemae Hospital, Osaka, Japan

**Keywords:** Cardiology, Diabetes

## Abstract

The coexistence of heart failure is frequent and associated with higher mortality in patients with type 2 diabetes (T2DM), and its management is a critical issue. The WATCH-DM risk score is a tool to predict heart failure in patients with type 2 diabetes mellitus (T2DM). We investigated whether it could estimate outcomes in T2DM patients with heart failure with preserved ejection fraction (HFpEF). The WATCH-DM risk score was calculated in 418 patients with T2DM hospitalized for HFpEF (male 49.5%, age 80 ± 9 years, HbA1c 6.8 ± 1.0%), and they were divided into the “average or lower” (≤ 10 points), “high” (11–13 points) and “very high” (≥ 14 points) risk groups. We followed patients to observe all-cause death for 386 days (median). We compared the area under the curve (AUC) of the WATCH-DM score for predicting 1-year mortality with that of the Meta-Analysis Global Group in Chronic Heart Failure (MAGGIC) score and of the Barcelona Bio-Heart Failure Risk (BCN Bio-HF). Among the study patients, 108 patients (25.8%) had average or lower risk scores, 147 patients (35.2%) had high risk scores, and 163 patients (39.0%) had very high risk scores. The Cox proportional hazard model selected the WATCH-DM score as an independent predictor of all-cause death (HR per unit 1.10, 95% CI 1.03 to 1.19), and the “average or lower” risk group had lower mortality than the other groups (p = 0.047 by log-rank test). The AUC of the WATCH-DM for 1-year mortality was 0.64 (95% CI 0.45 to 0.74), which was not different from that of the MAGGIC score (0.72, 95% CI 0.63 to 0.80, p = 0.08) or that of BCN Bio-HF (0.70, 0.61 to 0.80, p = 0.25). The WATCH-DM risk score can estimate prognosis in T2DM patients with HFpEF and can identify patients at higher risk of mortality.

## Introduction

Heart failure (HF) is one of the major complications of diabetes mellitus (DM) and is strongly associated with the prognosis of diabetic patients. HF is observed in up to 22% of diabetic patients, and patients with DM or prediabetes have a two to four times higher risk of developing HF than those without them^1–4^. HF is also the strongest risk factor for death among cardiovascular or renal complications in patients with newly diagnosed type 2 diabetes mellitus (T2DM) ^1,3,5^. On the other hand, DM is present in 20 to 40% of HF patients^4^. Diabetic patients have comorbidities such as hypertension, hypertension, atrial fibrillation, anemia, iron deficiency, serum potassium disturbances, frailty, and CKD more frequently^6–8^. and concomitant DM increases mortality and hospitalization in patients with HF^1–4,9^. HF with preserved ejection fraction (HFpEF) is as common as HF with reduced ejection fraction (HFrEF) in T2DM patients, and the impact of DM on cardiovascular death or HF hospitalization is greater in HFpEF than in HFrEF^9^.

Whereas most glucose-lowering agents did not reduce the incidence of HF in T2DM patients in randomized control trials^10^, and SGLT2 inhibitors can reduce HF incidence, leading to fewer MACE and cardiovascular death in patients with T2DM^11–13^. SGLT2 inhibitors reduce cardiovascular death and HF hospitalization in both HFrEF^14,15^ and HFpEF^16,17^ patients with or without DM. Guidelines recommend empagliflozin or dapagliflozin in patients with T2DM and LVEF > 40% (HF with mildly reduced EF and HFpEF) to reduce the risk of HF hospitalization or CV death^6,10^. However, SGLT2 inhibitors are more expensive than traditional treatments. Guidelines recommend that DM treatment regimens need to be continuously reviewed for efficacy, side effects, and burden, and in some cases, reduction or discontinuation of medication should be considered for several reasons, including cost^10^. Patients suitable for expensive treatments should be appropriately selected.

For the risk stratification of patients with T2DM and HF, prediction of functional and clinical outcomes is as important as prediction of HF development. The WATCH-DM risk score is a novel risk score for predicting incident HF hospitalization in T2DM patients without baseline HF, using readily available clinical, laboratory, and electrocardiographic (ECG) variables^18^. Most of these variables are relevant to heart failure, and we hypothesized that this score might be associated with severity of disease and clinical outcomes in patients with established heart failure. Thus, we investigated whether the WATCH-DM score can predict prognosis in T2DM patients hospitalized with acutely decompensated HFpEF. We also compared its ability to predict all-cause mortality with other established risk scores.

## Methods

### Study population

We retrospectively analyzed the data from the Prospective Multicenter Observational Study of Patients with Heart Failure with Preserved Ejection Fraction (PURSUIT-HFpEF), a multicenter, observational study enrolling consecutive patients hospitalized with acute decompensated HFpEF (left ventricular ejection fraction (LVEF) ≥ 50%) in 31 collaborating hospitals [UMIN-CTR ID: UMIN000021831]. Details of the entry criteria and data collection have been described elsewhere^19^. Briefly, patients admitted with acutely decompensated HFpEF were registered, and their clinical data, including medications, laboratory tests, and ECG, were collected on admission, at discharge, and 1 year after discharge. Acutely decompensated HFpEF was diagnosed based on the following criteria: (1) clinical symptoms and signs according to the Framingham Heart Study criteria, (2) LVEF on admission ≥ 50%, and (3) serum N-terminal pro-B type natriuretic peptide (NT-proBNP) ≥ 400 pg/mL or brain natriuretic peptide ≥ 100 pg/mL. T2DM was diagnosed based on clinical history or on fasting plasma glucose and/or hemoglobin A1c (HbA1c) during hospitalization based on the Japanese Clinical Practice Guideline for Diabetes 2019^20^. Oral glucose tolerance testing was not mandatory for the present study.

This study was conducted in accordance with the Declaration of Helsinki. The study protocol was approved by each corresponding hospital’s Ethics Committee. Informed consent was obtained from each patient by one of the investigators before the study.

### Data collection

We collected patient data, including risk factors and history of major comorbidities such as DM, hypertension, dyslipidemia, smoking, chronic kidney disease (CKD), history of HF hospitalization, prior myocardial infarction, prior stroke and malignancy, and history of percutaneous coronary intervention or coronary artery bypass graft (CABG). Blood tests, standard 12-lead ECG recording, chest radiography, and echocardiography were performed immediately after admission, before discharge, and 1 year after discharge. LVEF was determined by either the Teichholz method or the modified Simpson’s technique on admission but only by Simpson’s technique at discharge and 1 year after discharge. Study patients were followed up by direct contact or telephone interview to observe all-cause death, which was a primary endpoint of the PURSUIT-HFpEF registry^19^. For patients whose survival information could not be determined by these means, the data from National Vital Statistics of Japan, which includes all death records in Japan reported by prefectural public health centers, were used with the permission of the Ministry of Health, Labor and Welfare in accordance with the Statistics Act in Japan.

### The WATCH-DM risk score and other heart failure risk scores

We calculated the WATCH-DM score as the sum of the scores obtained from the following factors^18^: age (0 to 6 points), body mass index (BMI, 0 to 5 points), systolic (0 to 3 points) and diastolic (0 to 2 points) blood pressure (BP), fasting plasma glucose (0 to 3 points), serum creatinine (0 to 5 points), high density lipoprotein cholesterol (HDL-c, 0 to 2 points), QRS width on ECG (0 or 3 points) and history of myocardial infarction (0 or 3 points) or CABG (0 or 2 points), all of which were measured or obtained at hospital discharge (Table 1). The original study estimated the risk of incident HF as very low (≤ 7 points), low (8–9 points), average (10 points), high (11–13 points) and very high (≥ 14 points)^18^. In the present study, we used the same risk classification was used tentatively to estimate the risk of HF outcomes. Due to the limited number of patients, we combined patients with very low to average risk as the “average or lower” risk group.

**Table 1:** The WATCH-DM risk score.

Age (years)	< 50	50–54	55–59	60–64	65–69	70–74	≥ 75
	0	1	2	3	4	5	6
BMI (kg/m^2^)	< 25	25–34	35–39	≥ 40			
	0	1	2	3			
Systolic BP (mmHg)	< 100	100–139	140–159	≥ 160			
	0	1	2	3			
Diastolic BP (mmHg)	< 60	60–80	≥ 80				
	2	1	0				
FPG (mg/dL)	< 125	125–199	200–299	≥ 300			
	0	1	2	3			
HDL-c (mg/dL)	< 30	30–59	≥ 60				
	2	1	0				
Serum Cr (mg/dL)	< 1.0	1.0–1.49	≥ 1.50				
	0	2	5				
QRS width (ms)	≥ 120	< 120					
	3	0					
Ischemic events	Prior CABG		Prior MI				
	2		3				

The WATCH-DM score was calculated as the sum of scores, which are shown in the bottom row corresponding to each factor.

*BMI* body mass index, *BP* blood pressure, *FPG* fasting plasma glucose, *HDL-C* high-density lipoprotein cholesterol, *Cr* serum creatinine, *CABG* coronary artery bypass grafting, *MI* myocardial infarction.

To compare the ability of the WATCH-DM risk score to predict 1-year mortality with other HF risk scores, we calculated the Meta-Analysis Global Group in Chronic Heart Failure (MAGGIC) risk score^21^ and the mortality risk using the Barcelona Bio-Heart Failure Risk (BCN Bio-HF) calculator^22^. We calculated the MAGGIC risk score as the sum of scores provided from the following factors: LVEF (0 point to all present patients with HFpEF), age (0 to 15 points), systolic BP (0 to 2 points), BMI (0 to 6 points), creatinine (0 to 8 points), New York Heart Association (NYHA) class (0 to 8 points), male sex (0 or 1 point), current smoker (0 or 1 point), DM (3 points for all present patients), diagnosis of chronic obstructive pulmonary disease (0 or 2 points), first diagnosis of heart failure in the 18 months (0 or 2 points), not on beta blockers (0 or 3 points) and not on renin–angiotensin–aldosterone blockers (0 or 1 point)^21^. We estimated 1-year mortality by the web-based BCN Bio-HF calculator (http://ww2.bcnbiohfcalculator.org/web/, accessed at September and October 2021) using age, sex, NYHA class, LVEF, serum sodium, estimated glomerular filtration rate (eGFR), hemoglobin, loop diuretic dose, beta blocker, angiotensin converting enzyme inhibitor/angiotensin-2 receptor blocker, and statin treatments^22^. We did not include biomarkers for the present analysis because NT-proBNP was not measured in some patients, and cardiac troponin T or ST-2 was not measured at all.

### Statistical analysis

Continuous variables are expressed as the mean and standard deviation or median [the interquartile range (IQR)]. Categorical variables are expressed as absolute frequencies or relative percentages. We made comparisons by one-way ANOVA for continuous variables, and the significance of differences among groups was calculated with the Bonferroni correction. Categorical variables were compared with Fisher’s exact test. For the correlation between WATCH-DM score and continuous or ordinal values, Spearman's rank correlation coefficient was calculated. A Cox proportional hazard model for all-cause death was constructed including factors that showed significant differences (p < 0.05) among the 3 risk groups, although factors that were included by or highly related to the WATCH-DM score were not included. Event-free survival analysis was performed using the Kaplan‒Meier method with the log-rank test for group comparisons. We compared hazard ratios per unit of the WATCH-DM score between subgroups using Wald test.

We constructed time-dependent receiver operating characteristic (ROC) curves of the risk scores for all-cause death at 1 year after hospitalization using the “time ROC” package (ver 0.4) for R. We compared the difference between the estimated area under the curves (AUC) of two risk scores for each time point, and the p value between them was obtained by calculating the variance of the difference using the independent and identically distributed (iid) representation of the AUC estimators. Youden’s J statistics was calculated as sensitivity + specificity − 1 for all points of an ROC curve using “cenROC” package (ver 2.0.0) for R^23^, and the maximum value of the index was used as a criterion for selecting the optimum cut-off point. All statistical analyses were performed using R (version 4.1.1) or R with a graphical user interface EZR (Saitama Medical Centre, Jichi Medical University, Japan).

### Ethics approval and consent to participate

This study was conducted in accordance with the Declaration of Helsinki. The study protocol was approved by the ethics committee of each hospital according to the Ethical Guidelines for Medical and Health Research Involving Human Subjects issued by the Ministry of Health, Labor and Welfare in Japan. The study was conducted in accordance with the Declaration of Helsinki, the Ethical Guidelines for Medical and Health Research Involving Human Subjects, and other current legal regulations in Japan. Informed consent was obtained from each patient by one of the investigators before the study.

## Results

### Patient characteristics

Among 1095 patients hospitalized for decompensated HFpEF and registered in PURSUIT-HFpEF between June 2016 and December 2020, 418 patients (38.2%) had T2DM. The mean age of the study patients was 80 ± 9 years, and 207 (49.5%) patients were male. The mean HbA1c was 6.8 ± 1.0%, and the mean BMI was 23.1 ± 4.4 kg/m^2^. Diabetes was diagnosed before hospitalization in 316 patients (75.6%), and it was newly diagnosed after hospitalization in 102 patients (24.4%).

### The Watch-DM score

**Figure 1:**
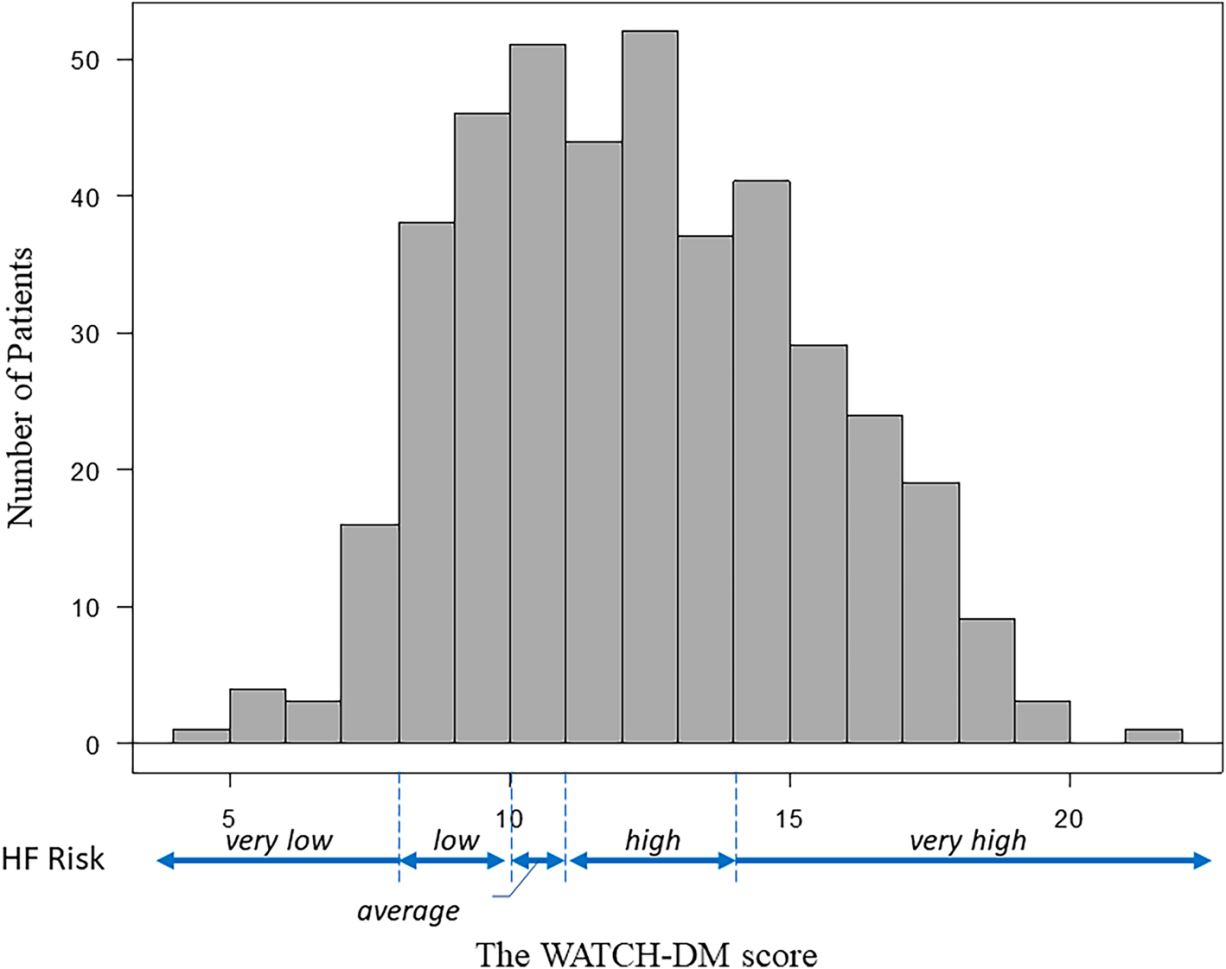
Distribution of the WATCH-DM scores in the study group. The number of patients with each score is shown as a bar. The heart failure (HF) risk was determined as the 5-year incidental HF risk in the original article^18^: ≤ 7 points very low; 8–9 points, low; 10 points, average; 11–13 points, high; ≥ 14 points, very high risk.

The mean WATCH-DM risk score in the study group was 12.8 ± 3.1 points, 108 patients (25.8%) had very low to average, 147 patients (35.2%) had high, and 163 patients (39.0%) had very high HF risk (Fig. 1). There were differences in clinical risk factors among groups, most of which were included in the WATCH-DM score: age (p < 0.0001), male sex (p = 0.003), BMI (p = 0.02), systolic (p = 0.02) and diastolic (p < 0.0001) BP, fasting plasma glucose (p = 0.003), serum creatinine (p < 0.0001) and LDL cholesterol (p = 0.002), whereas no difference was found in HbA1c (p = 0.70) and HDL cholesterol (p = 0.07) (Table 2).

**Table 2:** Patient characteristics.

Heart failure risk	Average or lower	High	Very high	P value
N	108	147	163	
WATCH-DM score	9.1 ± 1.1	12.0 ± 0.8^¶^	16.0 ± 1.7^¶,||^	< 0.001
Age, years	76.6 ± 11.2	81.1 ± 7.8^¶^	81.5 ± 6.8^¶^	< 0.001
Male gender, %	39.8	45.6	59.5^†^	0.003
BMI, kg/m^2^	22.3 ± 4.1	22.8 ± 4.5	23.8 ± 4.3*	0.02
Hypertension, %	82.4	91.2	93.9*	0.009
Dyslipidemia, %	41.7	54.4	67.5*	0.0002
Smokers, %	42.6	36.7	46.6	0.21
Chronic kidney disease, %	59.3	83.6^¶^	98.8^¶^	< 0.001
History of atrial fibrillation, %	42.5	40.1	33	0.23
Systolic BP, mmHg	118 ± 17	123 ± 18	123 ± 20*	0.02
Diastolic BP, mmHg	70 ± 12	65 ± 12	63 ± 13	< 0.001
Creatinine, μmol/L	77.8 ± 26.5	102.5 ± 45.1	225.4 ± 192.7	< 0.001
Estimated GFR, mL/min/1.73m^2^	57.3 ± 14.9	45.4 ± 15.0^¶^	26.0 ± 14.0^¶,||^	< 0.001
Fasting plasma glucose, mmol/L	6.38 ± 1.6	7.2 ± 2.5*	7.4 ± 2.7^‡^	0.003
HbA1c, %	6.8 ± 1.0	6.8 ± 0.7	6.9 ± 1.1	0.70
Triglyceride, mmol/L	1.22 ± 0.50	1.33 ± 0.61	1.49 ± 0.56	0.32
HDL-c, mmol/L	1.14 ± 0.31	1.11 ± 0.34	1.06 ± 0.36	0.08
LDL-c, mmol/L	2.51 ± 0.70	2.46 ± 0.80^‡^	2.20 ± 0.75^‡^	0.002
Medication at discharge				
Antiplatelets, %	18.5	33.3*	54.6^¶,||^	< 0.001
Anticoagulants, %	68.5	55.8	47.9^‡^	0.003
Statins, %	35.5	43.5	53.4*	0.01
Diuretics, %	82.4	85	84	0.85
RAAS inhibitors, %	53.7	59.9	55.8	0.60
β blockers, %	57.4	49.0	58.3	0.22
MRA, %	48.1	38.1	35	0.09
Digoxin, %	6.5	2.0	0.6	0.01
Calcium channel blocker, %	48.1	55.8	63.2	0.048
Oral glucose-lowering drugs, %	49.1	53.1	58.1	0.32
Biguanide, %	17.6	8.2	1.8^¶,§^	< 0.0001
Sulfonylurea, %	7.4	10.9	6.1	0.32
DPP-4 inhibitors, %	35.2	43.5	47.2	0.14
SGLT2 inhibitors, %	9.3	10.9	19.8	0.03
Insulin, %	9.3	7.5	18.4^§^	0.01
GLP-1 receptor agonists, %	1.9	0.7	3.1	0.35

The smokers included former and current smokers.

*BMI* body mass index, *BP* blood pressure, *GFR* glomerular filtration rate, *HbA1c* hemoglobin A1c, *HDL-c* high density lipoprotein cholesterol, *LDL-c* low density lipoprotein cholesterol, *RAAS* renin–angiotensin–aldosterone system, *MRA* mineralocorticoid receptor antagonist, *DPP-4* dipeptidyl peptidase-4, *SGLT2* sodium glucose cotransporter 2, *GLP-1* glucagon-like peptide-1.

*p < 0.05, ^†^p < 0.01, ^‡^p < 0.005, ^¶^p < 0.001 vs. the average or lower risk group, ^§^p < 0.05, ^||^p < 0.001 vs. the high-risk group.

NT-proBNP was measured in 359 patients (85.9%), and it was correlated with WATCH-DM score (ρ = 0.33, p < 0.0001). The “very high” risk group had a higher NT-proBNP value (1638 [774, 4848] ng/L) than the “average or lower” (696 [400, 1442] ng/L, p = 0.002) and the “high” risk (836 [386, 1665] ng/L, p = 0.003) groups.

There were differences in the rate of prescription of antiplatelets, anticoagulants, statins, digoxin, and calcium channel blockers among the risk score groups. Among diabetes medications, there were significant difference in the prescription of biguanides, sodium-glucose cotransporter 2 (SGLT2) inhibitors, and insulin.

Table 3 demonstrates echocardiography parameters measured at discharge. Left ventricular diameters were significantly higher in patients with very high risk than those with average or lower risk. They also had larger left ventricular mass index than those with average or lower risk and higher E/e′ ratio than other two groups, whereas no differences were observed in other diastolic parameters.

**Table 3:** Echocardiography parameters at discharge.

Heart failure risk	Average or lower	High	Very high	P value
LVDd, cm	4.6 ± 0.6	4.6 ± 6.5	4.8 ± 0.6*	0.02
LVDs, cm	3.0 ± 0.5	2.9 ± 0.5	3.1 ± 0.6*	0.01
Ejection fraction, %	59.2 ± 6.8	60.7 ± 7.7	60.7 ± 8.8	0.32
IVS thickness, cm	1.0 ± 0.2	1.0 ± 0.2	1.1 ± 0.2*	0.009
PW thickness, cm	1.0 ± 0.2	1.0 ± 0.2	1.1 ± 0.2	0.06
Left ventricular mass index, g/m^2^	103.0 ± 29.2	105.9 ± 32.0	115.1 ± 28.5^†^	0.003
Left atrial diameter, cm	4.3 ± 0.7	4.4 ± 0.8	4.5 ± 0.7	0.11
Left atrial volume index, ml/m^2^	50.8 ± 21.2	54.6 ± 26.0	50.9 ± 18.6	0.34
E/A ratio	0.95 ± 0.49	0.96 ± 0.60	0.98 ± 0.50	0.92
E/e’ ratio	13.0 ± 5.8	14.5 ± 5.4	15.6 ± 6.6^‡,¶^	0.004
TR-PG, mmHg	27.7 ± 9.0	27.6 ± 9.2	30.0 ± 11.3	0.11

*LVDd* end-diastolic left ventricular diameter, *LVDs* end-systolic left ventricular diameter, *IVS* intraventricular septum, *PW* posterior wall, *TR-PG* tricuspid regurgitation pressure gradient.

*p < 0.05, ^†^p < 0.01, ^‡^p < 0.005 vs. the average or lower risk group, ^¶^p < 0.05 vs. the high-risk group.

### Clinical outcomes and the WATCH-DM score

During the follow-up period of 386 [IQR 221 to 729] days, 77 patients (18.4%) died. We constructed a Cox proportional hazard model using sex, history of hypertension, CKD, dyslipidemia, LDL-cholesterol and the WATCH-DM score, which were significantly different among the 3 risk groups (Table 2), for all-cause mortality. We did not include age, BMI, systolic/diastolic BP, serum creatinine, or fasting plasma glucose because these factors were included in the WATCH-DM score. We did not include eGFR because creatine was used in the WATCH-DM score, while history of CKD was included in the analysis.

The Cox proportional hazard model selected the WATCH-DM score as an independent predictor for all-cause death (hazard ratio (HR) per unit 1.10, 95% confidence interval (CI) 1.01 to 1.20, p = 0.006), together with dyslipidemia and CKD (Table 4). When the classification by the risk score was used for analysis instead of WATCH-DM score, it was selected as the only significant predictive factor (HR 1.52, 95% CI 1.07 to 2.1, p = 0.02), whereas dyslipidemia (p = 0.06) and CKD (p = 0.10) were not. The adjusted HR in the “very high risk” group, with the “average or lower” risk group as reference, was 2.29 [95% CI 1.05 to 5.01, p = 0.04], while the HR in the “high risk” was not statistically significant (HR 1.69 [95% CI 0.79 to 3.60, p = 0.17]).

**Table 4:** Cox proportional hazard model analysis for all-cause mortality.

	Hazard ratio [95% confidence interval]	P values
Gender	1.311 [0.787–2.184]	0.298
Hypertension	0.566 [0.272–1.178]	0.128
Dyslipidemia	0.590 [0.35–0.998]	0.049
CKD	1.703 [1.002–2.895]	0.049
LDL-c	0.999 [0.989–1.008]	0.749
WATCH-DM score	1.102 [1.009–1.203]	0.031

*CKD* chronic kidney disease, *LDL-c* low-density lipoprotein cholesterol.

**Figure 2:**
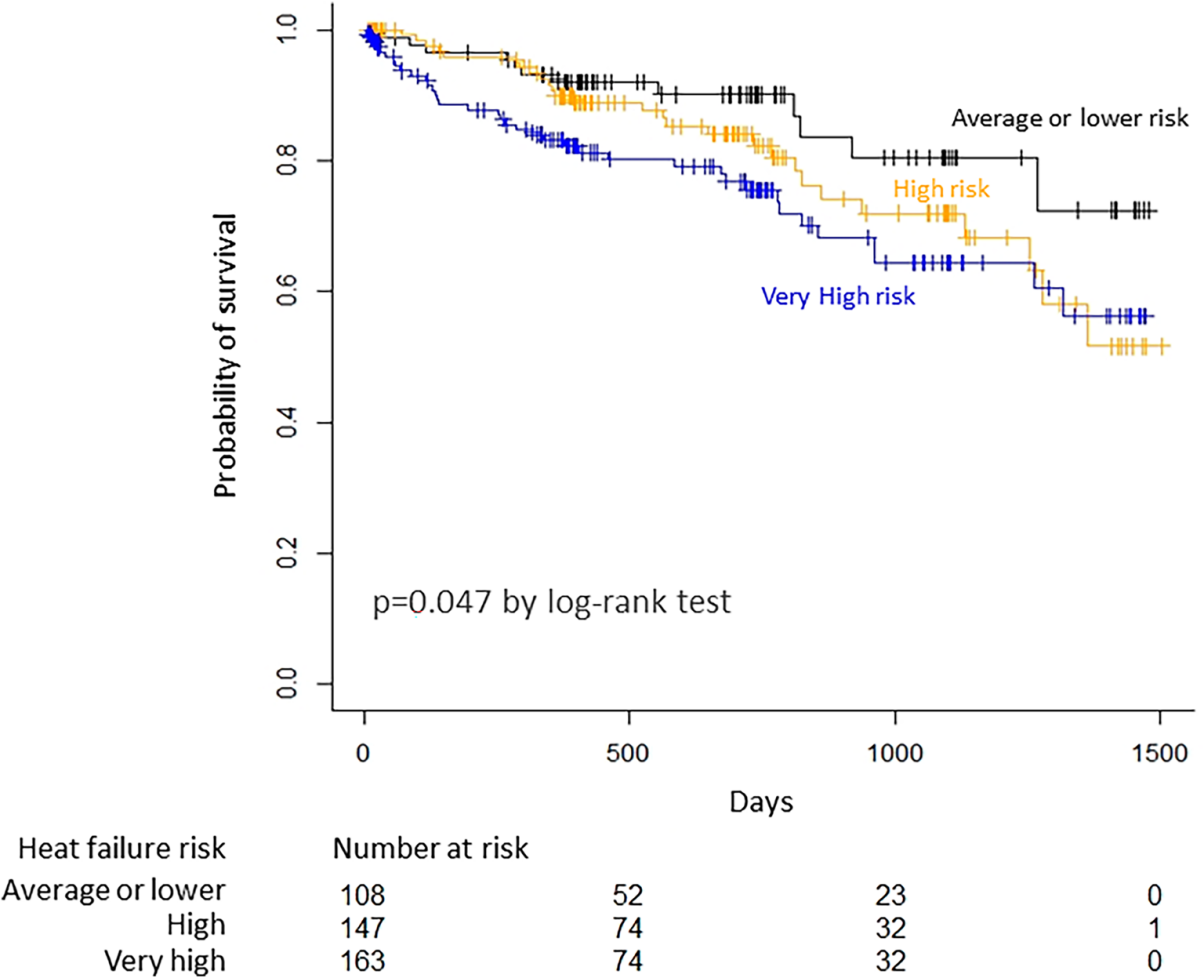
Kaplan‒Meier curves for all-cause mortality. Death was observed in 12 patients with average or lower risk, in 27 patients with high risk and in 38 patients with very high risk during follow-up period (median 386 days, IQR 221 to 729 days). There were significant differences in mortality among the three groups (p = 0.047 by log-rank test). Patients with very high risk (≥ 14 points) had a higher mortality than those with average or lower risk (≤ 10 points, p = 0.043).

Kaplan‒Meier curves showed the difference in all-cause mortality among the 3 groups (p = 0.047 by log-rank test), and the “very high” risk group had higher mortality than the “average or lower” risk group (p = 0.043) (Fig. 2).

**Figure 3:**
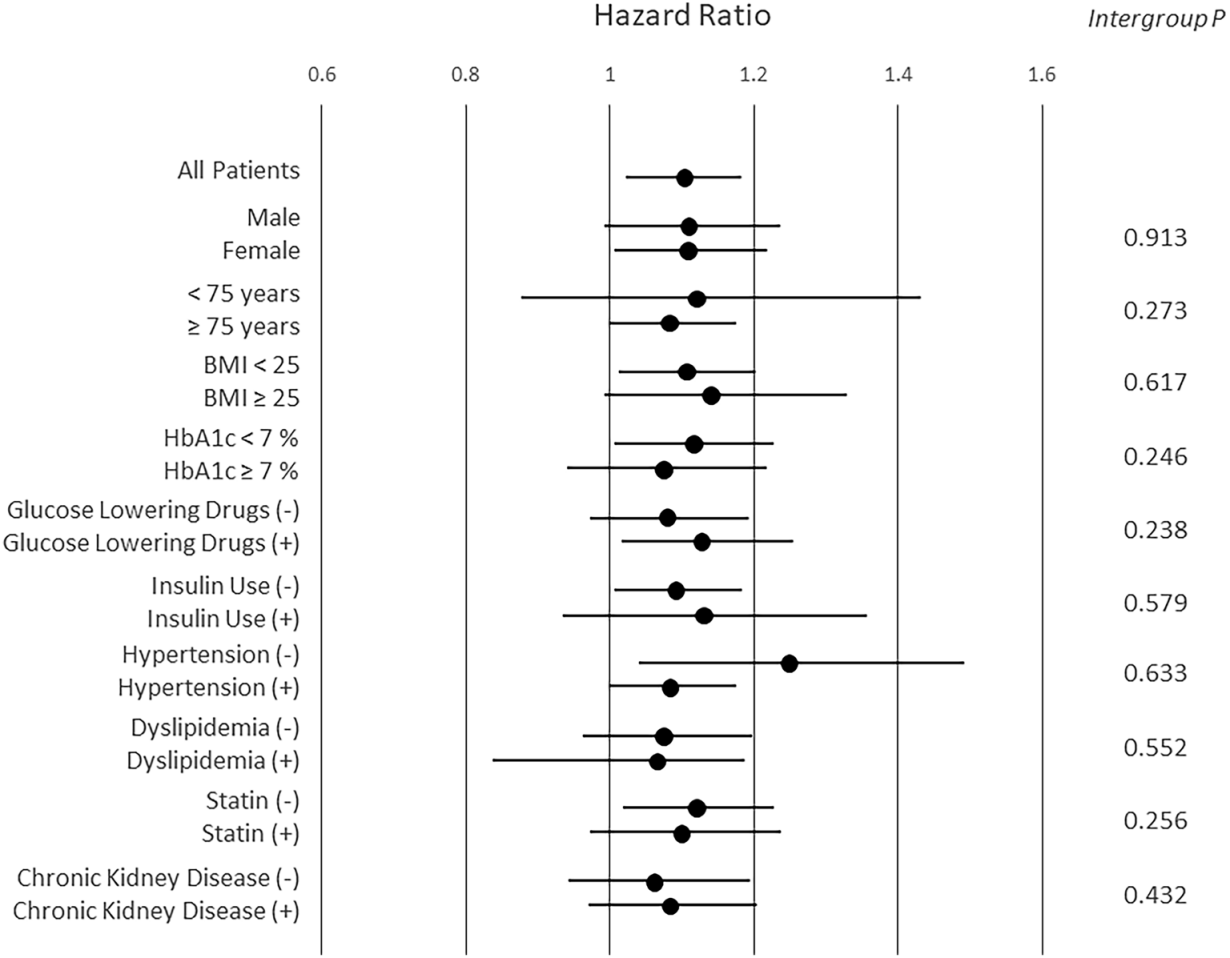
Subgroup analysis of hazard ratio per unit of the WATCH-DM risk score. Hazard ratios for all-cause mortality per unit of the WATCH-DM score were compared between subgroups based on age, gender, BMI, glycemic control, use of glucose lowering drugs, insulin, or statin, and risk factors. There was no significant difference between all subgroups.

No significant difference was observed in hazard ratios between subgroups based on age, gender, BMI, glycemic control, risk factors, and use of statin, glucose lowering drugs, or insulin (Fig. 3).

### Comparison of the WATCH-DM score and the other risk scores

**Figure 4:**
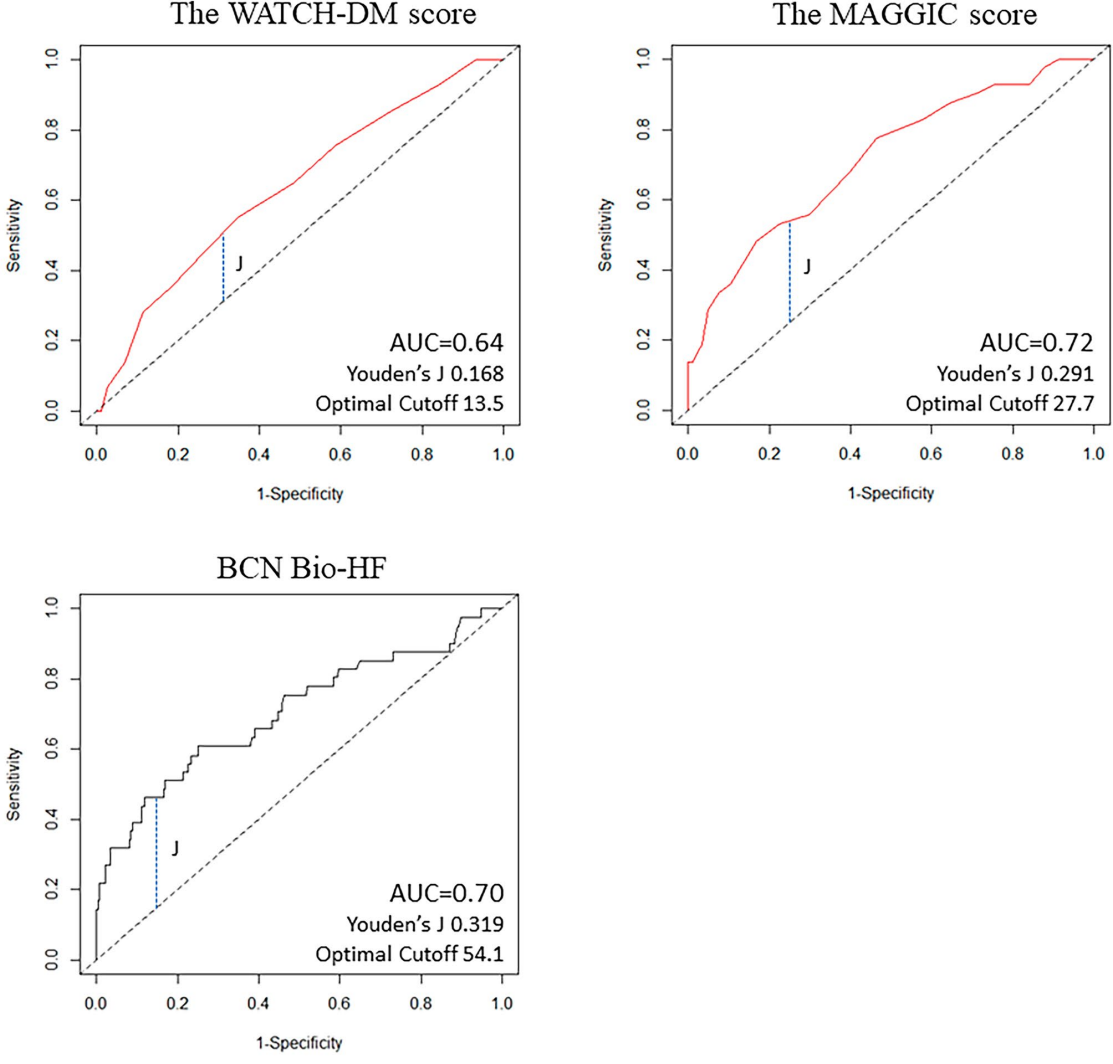
Time-dependent ROC curves for the prediction of 1-year mortality. Time-dependent ROC curves of the WATCH-DM score (top, left), the MAGGIC score (top, right) and BCN Bio-HF (bottom) for all-cause death at 1 year after hospitalization. Vertical dotted lines (J) indicated maximum value of Youden's J statistics. The areas under the curve (AUCs) of the WATCH-DM score and MAGGIC score were 0.64 (95% CI 0.45 to 0.74) and 0.72 (95% CI 0.63 to 0.80), respectively. The AUC of BCN Bio-HF was 0.70 (95% CI 0.61 to 0.80), which was not different from that of the WATCH-DM risk score (p = 0.25).

The MAGGIC score in the study group was 25.7 ± 5.6, and it was significantly correlated with WATCH-DM (ρ = 0.395, p < 0.001) (Fig. 4). The AUC of the WATCH-DM for all-cause death at 1 year was 0.64 (95% CI 0.45 to 0.74). The AUC of the MAGGIC score was 0.72 (95% CI 0.63 to 0.80), and there was no difference in AUC between the two scores (p = 0.08) (Fig. 4). The AUC for 1-year mortality prediction by the BCN Bio-HF calculator was 0.70 (95% CI 0.61 to 0.80), which was not different from that of the WATCH-DM risk score (p = 0.25). The maximum Youden’s J statistics for the WATCH-DM score, the MAGGIC score, and BCN Bio-HF was 0.168, 0.291 and 0.319, respectively. The optimal cutoff value of the WATCH-DM score determined by Youden’s J statistics was 13.5, which corresponded to a score that distinguished the “very high” risk group from the others.

## Discussion

We investigated the ability of the WATCH-DM risk score to predict all-cause mortality in 418 T2DM patients hospitalized for decompensated HFpEF. During the follow-up period of 386 (median) days, all-cause death was observed in 77 patients, and the WATCH-DM score was selected as an independent predictor for all-cause death (HR per unit 1.10, 95% CI 1.01 to 1.20, p = 0.006), along with dyslipidemia and CKD. Significant differences in all-cause mortality were observed among the “average or lower’’ (≤ 10 points), the “high” and the “very high” (≥ 14 points) risk groups (p = 0.047 by log-rank test), and the very high risk group had higher mortality than the “average or lower" risk group (p = 0.043). The AUC for the prediction of all-cause mortality by the WATCH-DM score was not different from that of the MAGGIC score (p = 0.08) and that by the BCN Bio-HF calculator (p = 0.25). These results indicate that the WATCH-DM score was associated with all-cause mortality in T2DM patients with HFpEF, and a 1-point increase of the WATCH-DM score was associated with a 10% increase in mortality.

This study was based on a large multicenter registry study of patients with decompensated HFpEF, the PURSUIT-HFpEF study, which enrolled more than one thousand patients from 31 collaborating hospitals (1 university hospital and 30 regional core centers)^19^. The PURSUIT-HFpEF study was unique in that it enrolled a heterogeneous and large number of patients with broad inclusion criteria and few exclusion criteria to provide an accurate understanding of real-world patients with HFpEF. Detailed clinical data were collected using an electronic data capture system integrated with the electronic medical record, and clinical outcomes were followed as closely as possible, even using the national death certification system^19^. Survival data were compared using statistically appropriate methods, including time-dependent ROC curve analysis. We believe that one of the strengths of this study was the highly reliable data collection, which well reflected a real-world HFpEF population.

The predictive value of the MAGGIC score and BCN Bio-HF in diabetic patients with HFpEF has not been previously reported. To our knowledge, this study is the first to demonstrate how well these risk scores can predict all-cause mortality and to directly compare their predictive values in this complicated patient population.

### The heart failure risk scores for diabetic patients

The management of heart failure in diabetic patients is more complicated than that in non-diabetic patients, and intensive glycemic control does not consistently correlate with improved clinical outcomes in HF patients with diabetes. While the risk of HF hospitalization tends to increase progressively with fasting blood glucose^24,25^, the association between HbA1c and mortality among patients with HF is consistently U-shaped, with the lowest mortality in individuals with HbA1c 7–8%^26–29^. HbA1c < 7% may be associated with worse prognosis in diabetic patients with HFpEF after adjustment for age, BMI, hemoglobin and NT-proBNP^30^.

Most of the risk scores for predicting HF prognosis have been derived from the data of HF patients with or without diabetes, and they may not adequately account for the influence of glycemic control. The presence of DM is used to calculate the MAGGIC score, but blood glucose or HbA1c is not included^21^. BCN Bio-HF does not use any factors related to DM or glycemic control^22^. The WATCH-DM score uses fasting plasma glucose for its calculation, reflecting the importance of metabolic factors in the development and prognosis of HF in diabetic patients.

The European Society of Cardiology guidelines for the management of cardiovascular disease in diabetic patients recommend that systematic survey for HF symptoms and/or signs of HF at each clinical encounter in all diabetic patients^6^. The WATCH-DM scores use factors that are commonly available in daily DM practice. Uniform assessment of the WATCH-DM score in the diabetes clinic would be useful in assessing the risk of incident HF in patients without HF and the prognostic risk in those with comorbid HF.

The present study did not show that the WATCH-DM score is superior to the MAGGIC score and BCN Bio-HF in predicting prognosis of T2DM patients with HFpEF. AUC of the WATCH-DM score was 0.65, which was classified as satisfactory but not optimal. Recently, Zhang et al. reported similar results on the predictive value of the WATCH-DM score in diabetic patients with HFpEF in a single-center study^31^. Their results showed a similar AUC value for the prediction of all-cause mortality (AUC = 0.67, 95% CI 0.59–0.74) to the present one, while it was lower than that for cardiovascular death (0.71, 95% CI 0.63–0.78). They reported that the WATCH-DM score did not stratify the risk of all-cause or cardiovascular mortality in non-diabetic patients with HFpEF^31^, suggesting the heterogeneity of HFpEF phenotypes between diabetic and non-diabetic patients and the need for more effective risk scores specified for HFpEF patients with T2DM.

### Improving prognostic prediction in T2DM patients with HFpEF

If WATCH-DM is not superior to other risk scores, are there ways to improve its predictive power?

The coefficients assigned to the variables within the WATCH-DM score were derived to predict the occurrence of incident HF regardless of LVEF, not for evaluating clinical outcomes in HFpEF. Among the present study patients, 108 patients (25.8%) had average or lower risk, while 5-year incident HF risk of incident HF was 1.1% for the very low-, 3.6% for the low-, and 4.7% for the average risk group^18^, which implied that the WATCH-DM score may underestimate the risk of HFpEF. While a linear relationship between BP and prognosis is observed in the general population, its relation may be inversed or J-shaped in patients with HF^32^. Systolic BP of 120–129 mmHg had the lowest cardiovascular event risk and < 120 mmHg was associated with a higher incidence of all-cause mortality in patients with HFpEF^33,34^. A U-shaped relationship was also observed between BMI and clinical outcomes including mortality in HFpEF^35–37^. Inclusion of these variables in a linear fashion may have reduced the prognostic value of the WATCH-DM score. Optimizing the coefficients for the variables may improve predictive value of the risk score, although this requires a much larger study cohort than the present study group.

The MAGGIC risk score and the BCN-Bio calculator include medications as factors for calculation. The WATCH-DM score does not include any medication as a factor, and which may be one of the reasons why the WATCH-DM score did not outperform other HF risk scores. Adding the use of SGLT2 inhibitors, which are recommended for both T2DM and HFpEF, would improve predictivity of the WATCH-DM score. The present data were collected before SGLT2 inhibitors were recommended for the treatment of HFpEF, and the number of patients taking SGLT2 inhibitors or GLP-1 receptor agonists was so small that their effects could not be properly analyzed.

Natriuretic peptide has a high prognostic value in diabetic patients as well as in HF patients. Left ventricular diastolic dysfunction is frequently observed in asymptomatic diabetic patients, and it can be effectively detected by increased natriuretic peptide^38^. Serial monitoring of NT-proBNP in T2DM patients may be useful to identify patients at highest risk of HF^39^, and guidelines recommend measurement of a natriuretic peptide in asymptomatic diabetic patients on at least a yearly basis to identify the earliest HF stages and implement strategies to prevent transition to symptomatic HF^2^. The addition of natriuretic peptide levels to the WATCH-DM risk score was associated with greater improvement in the prediction of the incident HF^40^. Prediction of clinical outcomes by the WATCH-DM score may be improved by addition of natriuretic peptide.

The TRS-HF_DM_ (Thrombolysis in Myocardial Infarction [TIMI] risk score for heart failure in diabetes) risk scores was recently developed to predict HF risk in T2DM patients^41,42^. While the TRS-HF_DM_ score was developed to predict HF hospitalization in patients with and without a prior HF history, it could predict incident HF events as well as the WATCH-DM among T2DM patients without previous HF hospitalization^43^.

Ceramides are associated with the development of diabetes and its complications including heart disease. The Ceramide risk score has been developed for clinical use based on ceramide concentrations and their ratios^44^, and it can predict major adverse cardiovascular events independent of coronary artery disease or conventional risk factors^45,46^. Elevated ceramide level are linked to insulin resistance^47^ which may be related to cardiac function in HF patients^48,49^. It should be investigated whether new risk scores such as the TRS-HF_DM_ score or the ceramide score could predict clinical outcomes in T2DM patients with HFpEF.

### Study limitations

The present study was a retrospective study from a registery database with a limited number of patients, and therefore, there could be some bias which could affect the present results. Oral glucose tolerance testing was not mandatory in the PURSUIT-HFpEF registry and DM may have been underdiagnosed, resulting in a higher WATCH-DM score in the study group. Patients with very high risk were more likely to receive antiplatelets and statins than other groups. The number of patients taking particular glucose-lowering drugs such as SGLT2 inhibitor or GLP-1 receptor agonists were so small that their effects could not be properly analyzed. Patients at higher risk tended to have lower rates of atrial fibrillation, though not statistically significant, and this may be due to selection bias.

Ethnic differences exist in the mortality and morbidity of T2DM^50^ and HFpEF^51–53^. The mean BMI was only 23.1 kg/m^2^, and the mean age was 80 years in the present study patients. Their characteristics were different from those of typical Western patients, and it is unclear whether the present results are applicable to patients outside of East Asia. A recent study demonstrated that treatment with semaglutide reduced body weight and improved symptoms and physical activity in patients with HFpEF and BMI ≥ 30 kg/m^2^, indicating the importance of BMI in the pathophysiology of HFpEF^54^. The low BMI in the study group has made the contribution of this important factor to the risk score almost negligible.

Difference in characteristics and outcomes of HF patients has also been observed between urban and rural areas^55,56^. All of the participating hospitals of the PURSUIT-HF study were located in a single urban region of Japan, which may introduce some bias in patient background.

Despite these study limitations, the present study suggested the possible role of the WATCH-DM score in the management of T2DM patients with HFpEF. The present study was a small-scale, retrospective proof-of-concept study. A large-scale, prospective study is required to establish the way to predict clinical outcomes in this challenging patient population.

## Data Availability

The datasets generated and/or analyzed during the current study are available from the corresponding author on reasonable request.
